# Greenfield investments projects in manufacturing and electricity: A network analysis.

**DOI:** 10.1371/journal.pone.0316647

**Published:** 2025-05-15

**Authors:** Gilson Geraldino Silva Jr, José Maria Ferreira Jardim da Silveira, Henrique Reichert

**Affiliations:** 1 Department of Economics, Federal University of Santa Catarina, Florianópolis, Santa Catarina, Brazil; 2 Institute of Economics, State University of Campinas, Campinas, São Paulo, Brazil; 3 Caravela Consulting, Florianópolis, Santa Catarina, Brazil; Universidad Mayor, CHILE

## Abstract

**Aim of the paper:**

Greenfield is, by definition, foreign direct investment that creates new production facilities in the host countries, which imply an addition of fresh foreign capital. This paper brings empirical evidence about Greenfield global investment projects using network analysis in two key sectors: manufacturing and electricity. Our main research question is: What is the Greenfield Global Web of Investment Projects? And related to this main question: What is the center-periphery positions? How did the 2008 crisis affect it? Our secondary questions are: What are the web network properties? What does it tell us about the spread of investment intentions?.

**Theoretical background:**

Network theory tell us, on one hand, that human decisions are made in the context of, and shaped by, web of interactions, and on the other, how dense network is, whether some groups are segregated and who sits in central positions, affects how information spreads and how people behave, and how linked agents interact with other linked agents, the outcomes ultimately depend on the entire network structure. This paper presents an original approach, combining different clustering methods in one figure. One is the Louvain without weights (once everything is measured in terms of indegree) using the concept of modularity. The other is positional analysis by blockmodeling.

**Data and results:**

Our data source is Financial Times (FT) Greenfield investments data base (2003–2016). FT is the most authoritative source of intelligence on real investment in the global economy, and the only source of Greenfield investment data that covers all countries and industries worldwide. The World Bank, Unctad, the Economist Intelligence Unit and more than 100 governments around the world as well as major corporations use the data as the primary source of intelligence on Greenfield investment trends. Our variables are investment projects intentions value and country origin by year. Our analytical technics are network indicators at exploratory level and blockmodel. We clear answer our research questions in the Analysis of the features of the Networks sections. We also highlight that i) the 2008 crisis had a deep impact on the networks equilibrium, ii) it was substantially deeper in electricity than in manufacturing, which suggests that manufacturing web of investment projects was more resilient to exogenous shock, iii) global intentions of investment cohesion and core-periphery relations increased after crises in both sectors. There are also some secondary, but important results: from the network indicators, there was more ties after crises, the network were smaller, the information reached easily, central countries importance in the expectation formation increased substantially in manufacturing but unchanged in electricity, the information to the central countries became shorter after crises, and the flow of expectation sent between dissimilar increases after crises.

## Introduction

Projects, intentions and believes are synonyms in this context. We have data about greenfield projects, so it reveals investor’s intentions or believes about the future in an uncertainty environment.

Believes formation under uncertainty is an old theoretical subject with few empirical evidences. Some earlier theoretical reference is [21,22] path breaking models about choice under uncertainty. This go throw probability and uncertainty in economic modelling debate as [19] sum up, the psychologically associated with uncertainty in [24,25] perspective, and some related evidence as [4,5,10,11,29]. A better understanding of heuristics and biases to which economic agents lead could improve judgments and decisions under uncertainty. The judgments must be compatible with the entire web of beliefs held by the individual [24]. Once humans are fundamentally a social species with interaction patterns that shape their behaviors, many human decisions are made in the context of and shaped by networks of interactions, so network analysis is a key tool to understand economic behavior [23]. Those agents play games in a network environment, a particular kind of strategic interaction that can be represented formally through a network, interaction matrix, or graph, whose applications includes social interaction, oligopoly and belief formation [12]. Psychological games [18] and the relationship between strategic interaction and individual believes in an institutional perspective [3] also help us understand this phenomenon. Network analysis and block modelling in Economics, particularly, are not new, back at least to 1990`s, and increase substantially in 2000’s and 2010’s. Nevertheless, we didn’t find any study using that methodologies to analyze Greenfield Foreign Direct Investment (by definition, investments that create new production facilities in the host countries, which imply an addition of fresh foreign capital, says [2,9,14,26,30,33]) intentions associated with foreign investors in two key sectors: Electricity, indispensable once without almost anything works, and Manufacturing, a high innovative industry that create high qualified jobs and it is connected with all others economic sectors in a way it is indispensable.

On the theoretical side, [19] sum up a long debate about probability and uncertainty in economic modelling, and remembered us that since the early days of probability theory there has been a distinction between probabilities that are given, as in a game of chance, and probabilities that are not given, as in an investment decision, but reflect a subjective degree of belief. If objective probabilities are not known, they could be replaced by subjective ones, so that problems of decision under uncertainty are reduced to problems of decision under risk. But it is not always clear how subjective beliefs should be formed. And in many economic problems it is not clear how probabilities should be defined.

In fact, the theory that results contains uncertainty, imperfect knowledge as the state of the world that will actually obtain in the future, but does not contain the “vagueness” we usually find psychologically associated with uncertainty. It sends us to [24] and their analysis about heuristics and biases in judgment under uncertainty. According to them, a better understanding of these heuristics and of the biases to which they lead could improve judgments and decisions in situations of uncertainty. The judgments must be compatible with the entire web of beliefs held by the individual. In doing so, a critique of expected utility theory as a descriptive model of decision making under risk is not a surprise, as well an alternative model, as [25] prospect theory.

On the empirical side, [4] used micro data from the West German manufacturing subset of the IFO Business Climate Survey to infer quarterly production changes at the firm level and combine them with production expectations over a quarterly horizon in the same survey to construct series of quantitative firm-specific expectation errors and find that, depending on the details of the empirical strategy, between 6% and 34% of firms systematically over or underpredict their one-quarter-ahead upcoming production. And [10] simulations based on 672 publicly traded U.K. manufacturing companies over the period 1972–1991 suggests that one standard deviation increase in their measure of uncertainty, like that which occurred after the 1973 oil crisis, September 11, 2001, and 2008 global financial crises, can split the impact effect of demand shocks on company investment. It means that firms will generally be less responsive to monetary and fiscal stimuli in periods of high uncertainty, which is important for policy-makers trying to respond to major shocks during periods of high uncertainty. At least, they argue we should highlight the role of sentiment as a potential driver of the business cycle. [29] argue that recent literature shows correlation between sentiment (considering both confidence and uncertainty) and economic activity, and points out three potential transmission mechanisms of sentiments to the real economy: animal spirits, self-fulfilling prophecies, and news and noise. They also identify new stylized facts based on international evidence strong enough to suggests the existence of a global factor or sizeable international spillovers of sentiment.

Blockmodeling in Economics, particularly, is not new, it back at least to 1990`s, and increase substantially in 2000’s and 2010’s. One example of network analysis and blockmodeling in Economics in 1990’s is [31] using global blockmodeling 1980’s algorithm to analyze the structure and dynamics of the global economy examining international trade networks in three different moments (1965, 1970, and 1980), a panel data with 63 countries in the three periods, and 15 types of trade commodities in 5 categories. A 2000’s example is [27], that also use network analysis of international trade, with pair-wise data trade flows between 53 countries measured in current U.S. dollars in 1965,1970,1980,1990 and 2000, close to [31]. And a 2010’s example is [20], based on a core–periphery perspective, analyze the service industry of stock photography in 37 countries.

In sum, there are a variety of expectations analysis, on one hand, and network, on the other, but, as far we know, any investment intentions network analysis, particularly for manufacturing and electricity. Notwithstanding, sometimes a shock is exogenous in some sectors but endogenous in others. Once 2008 was a financial crise, it was an endogenous shock to the financial market, but it was an exogenous one for the two sectors we are considering.

In doing so, our main research question is: What is the Greenfield Global Web of Investment Projects? And related to this main question: What is the center-periphery positions? How did the 2008 crisis affect it? Our secondary questions are: What are the web network properties? What does it tell us about the spread of investment intentions?

## Materials and methods

### Data base

According to [32], the FDI Markets is a database of The Financial Times Ltd. It is the most authoritative source of intelligence on real investment in the global economy, and the only source of Greenfield investment data that covers all countries and industries worldwide. The World Bank, Unctad, the Economist Intelligence Unit and more than 100 governments around the world as well as major corporations use the data as the primary source of intelligence on Greenfield investment trends. FDI Markets is also used for recent academic purposes, as [13] global R&D location analysis between 2003–2014 and [1] foreign R&D investments investigation between 2003–2016. We work on that information and set-up 2003–2008 and 2009–2016 web of Greenfield investment. Investment projects intentions in manufacturing was, in average, US$ 120,53 US$ million in 2003–2008, and US$ 112,49 US$ million in 2009–2016, or 6,67% less in average. In electricity, it was US$ 495,80 US$ million in 2003–2008 and US$ 448,80 US$ million in 2009–2016, or 9,48% less in average. All values in 2016 base. Manufacturing projects had origin in 124 countries and electricity in 100.

### Network indicators

The network is built taking into account the aggregation of directional intentions of investors from one country to another. It allows a bidirectional flow. For instance, investors from Japan in the manufacturing sector declare their intentions to Vietnam each period. The sum of declarations generates an arc (directional) pointing to the receiver. The same processes from Vietnam to Japan generate another arc pointing to Japan. [16] explain how to obtain an Adjacent Matrix that represents the ensemble of these relations.

The flows can be weighted or not. In many procedures, for example, block modeling, the calculation only considers the link’s existence. The weights, represented by the amount of each flow, contribute to evaluating the importance of each country. The amount of money for the projects is represented by the size of the vertices in Figs 2 and 3.

**Fig 1:**
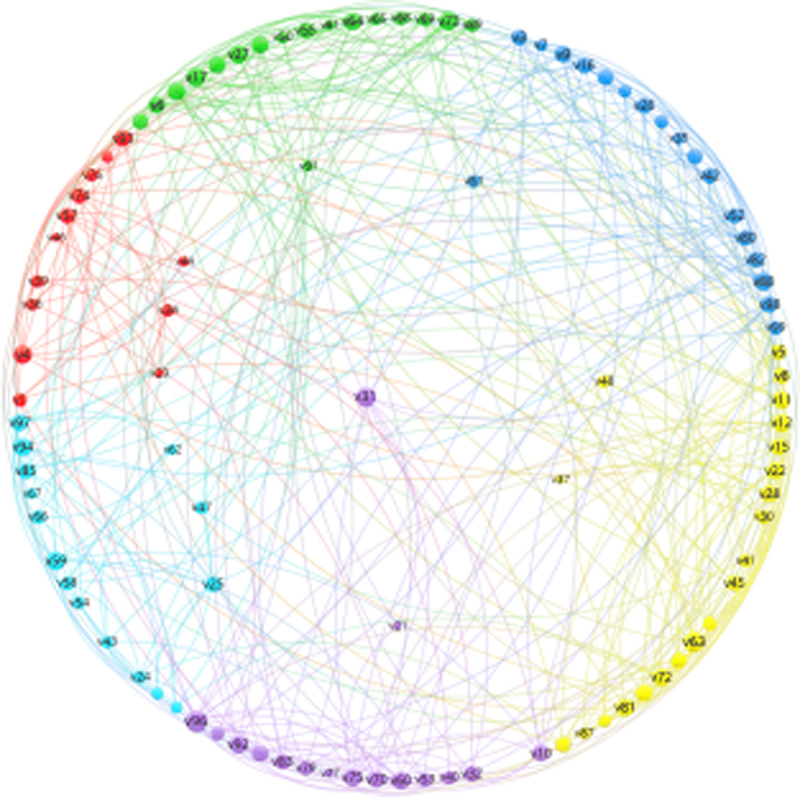
A Simulated Network with countries organized in two lawyers: Center-Sub Center and Periphery and Clusters. Source: Author’s elaboration from FT data base.

**Fig 2:**
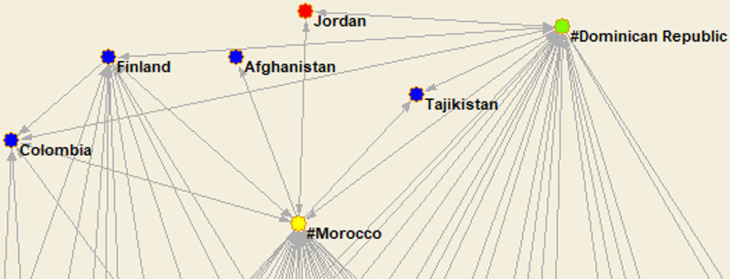
An illustration of arcs in the original network after applying the two clustering procedures. Source: Author’s elaboration from FT data base.

**Fig 3:**
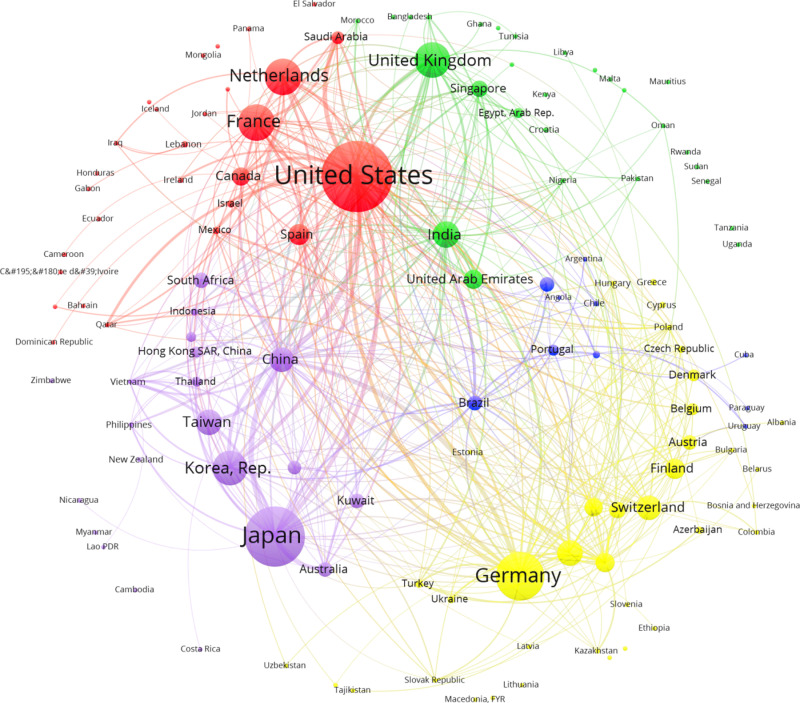
Manufacturing investors’ intentions networks. Before 2008. Source: Author`s elaboration using FT data base.

About network properties, some of them give us important information about agent’s economic behavior. One is density. It relates to diffusion and contagion. Denser networks, in terms of average numbers of connections per node lead to more extensive diffusion or contagion, ceteris paribus. It leads to more interactions and greater basic reproduction numbers (holding fixed the probability of transmission via any given interaction), according to [23]. But there are more networks properties as number of lines, average degree, hierarchy, centralization, betweenness and assortativity ([16]).

Following [16], we consider indicators since the exploratory level as the number of lines (a tie between two vertices in a network, vertices is a set of vertex, the smallest unit in a network), cohesion indicators as density (the number of lines in a simple network, expressed as a proportion of the maximum possible number of lines), average degree (degree of a vertex is the number of lines incident with it) and hierarchy (a data object for classifying vertices if a vertex may belong to several classes), and center periphery indicators as betweeness centralization (the variation in the betweeness centrality of a vertice divided by the maximum variation in betweeness centrality scores possible in a network of the same size; betweeness centrality of a vertex is the proportion of all geodesics between pairs of other vertices that include this vertex; and geodesic is the shortest path between two vertices), degree centralization (the variation in the degrees of vertices divided by maximum degree variation that is possible in a network of the same size), and assortativity or homophily (the preference of vertices to attach to other vertices that are similar to them according to a numeric property).

Those indicators have an intuition behind, also according to [16]. Density (d) is inversely related to network size because the number of possible lines increases rapidly with the number of vertices, so larger networks tend to be less dense, and d = 0.15 means that only 15% of all possible arcs are present. Degree of a vertex informs how easily an information can reach a person. In this sense, indegree (in) of a vertex is the number of arcs it receives, and outdegree (out) of a vertex is the number of arcs it sends. Hierarchy is particularly useful for a hierarchical clustering of vertices in which units are subdivided into more and more homogeneous subsets. More centralization means that shortest path between two vertices decrease. Betweeness is an alternative to centrality, it inform how a person, company of country is more central if he is more important as an intermediary in the communication network, how crucial he is in the transmission of information through a network, how many flows of information are disrupted (or must take longer detours) if a person stops passing on information (or disappear from the network), in what extent may a someone control the flow of information due to his position in a communication network, or tell us the extended someone is needed as a link in the chains of contacts that facilitated the spread of information within the network. At least, assortativity or homophily means that similar interact more than dissimilar.

## Clustering methods in one figure

[29] pioneered the discussion on countries’ economic relations, represented by networks, introducing the positional analysis. In their view, a rigid division in center-periphery misrepresents economic ties between countries regarding economic flows. In the present paper, the network considers all intentions of the investments between two countries, with directional links, which means arcs in manufacturing and electricity, two key sectors in any modern economy. Electricity is indispensable once without almost anything works. Manufacturing nowadays is high innovative, create high qualified jobs, and it is connected with all others economic sectors in a way it is indispensable.

The paper presents an original approach, combining different clustering methods in one figure. One is the Louvain without weights (once everything is measured in terms of indegree) using the concept of modularity. The process looks for a better configuration of the clusters based on the links between countries, discounting the “trivial links.” For instance, if a small or medium-sized country is linked to the USA in the manufacturing sector, this link receives less weight than a link with another small country. The algorithm looks for the cuts given the network’s cluster configuration. [8] and [15] explains the algorithm’s functioning in detail.

The other is the positional analysis by blockmodeling. One of the main challengers in economic analysis nowadays is sum up larger data base as a clear and useful set of information. In network analysis context block modeling reduces a large and potentially incoherent network to a smaller comprehensible structure that can be interpreted more readily [6,7]. It is a single technique that is able not only to detect different kind of structure but also to ascertain cohesion and core-periphery structures [16].

The paper’s methodological choice is the “direct” approach, which uses the concept of equivalence instead of similarity or dissimilarity, used in “indirect” cluster analysis [7]*.* Intuitively, it means that a country could be placed in the same cluster as another due to an equivalent role in the network, and an arc does not necessarily link countries of the same cluster. In other words, they fulfill the same role in the blockmodeling map.

As the network indicator structure, block model also has an intuition behind. It is a flexible matrix-based method for analyze and visualize networks, capable of detecting cohesion, core-periphery structures, and ranking, but not replace the indicators in section about network indicators. It assigns the vertices of a network to classes, and it specifies the permitted types of relation within and between classes.

Fig 1 presents a simulated network with features similar to “real” networks related to the manufacturing and electricity sectors. The likelihood implies applying the same “pre-specified block model” we use to study the manufacturing and electricity sectors. The positional approach contributes to organizing the disposition of the countries in the network [16,17]. In the center of Fig 1, we can find the “core” or center (only one country is found there). Around the center, the sub-center or intermediate center, with almost 12 countries, and, in the external part, the periphery. The link between the position and clusters is clear once a country in the intermediate region is linked preferentially to countries of the same cluster, including periphery countries. Finally, a country’s peripheral position means it has links with countries outside the periphery. In the prespecified block model, links between peripherical countries are a mistake, generating a penalty in the ex-post goodness-of-fit evaluation [16]. To a better visualization of the network, as in Figs 2 and 3, we use the VOS viewer feed by the results of the Pajek version 5.17’analysis [28].

**Fig 4:**
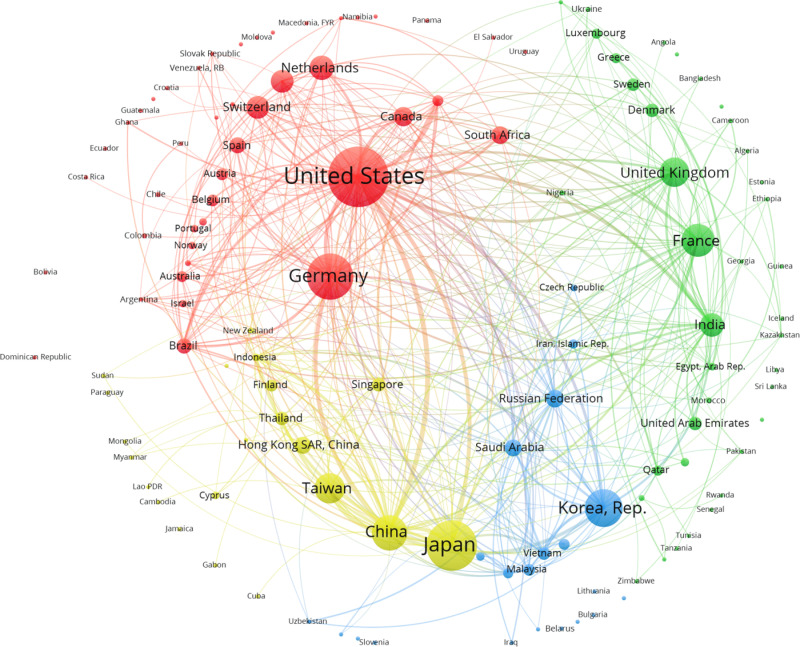
Manufacturing investors’ intentions networks. After 2008 Source: Author`s elaboration using FT data base.

**Fig 5:**
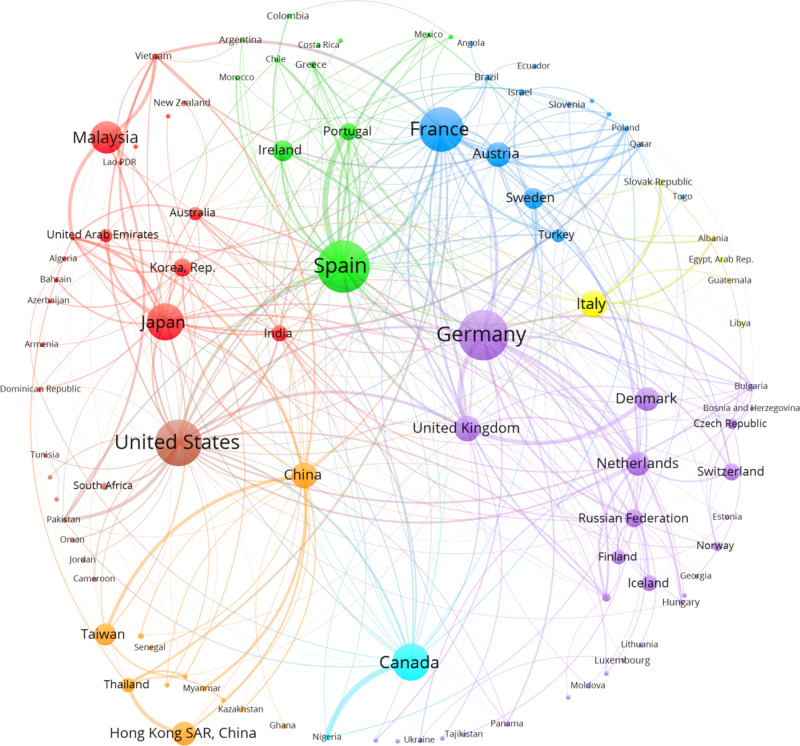
Electricity investors’ intentions networks. Before 2008. Source: Author`s elaboration using FT data base.

**Fig 6:**
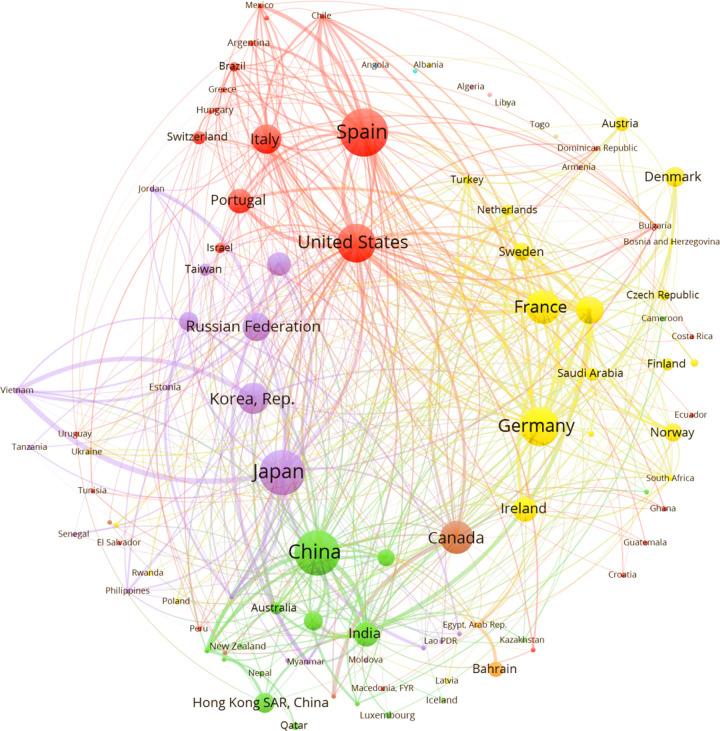
Electricity investors’ intentions networks. After 2008. Source: Author`s elaboration using FT data base.

To better interpret Figs 3 to 6 two aspects need to be clarified: a) The size of the vertices represents the importance of the country as investor and receiver of intentions of investment; b) the links between two countries (diad) represent a condensed value of the flow between them, the thicker, bigger the flow. This is only to have a clear graph. In fact, the original graph consists of a beam of arcs pointing to the receiver (e.g., Japan to Vietnam, and vice versa, if that is the case).

Fig 2 illustrates a segment of the original network (after the clustering procedures) showing the presence of bilateral arcs (e.g., Jordan investing in Marrocco and vice versa) and a single arc (the case of Finland investing in Colombia). Fig 2 also illustrates how confusing the graphs would be without the technique of aggregation flows and representing the result by the thickness of the arcs.

From a formal view, Block model is based on network, cluster and block definitions [6,7]. Let E = {X_1_, X_2_,…, X_n_} be a finite set of units. The units are related by binary relations R_t_ ⊆ E × E, t = 1,…, r, r ≥ 1 which determine a network N = (E, R_1_, R_2_,…, R_r_). We restrict our discussion to a single relation R described by a corresponding binary matrix R = [r_ij_]n × n where r_ij_ = 1 if X_i_RX_j_; otherwise r_ij_ = 0. In some applications r_ij_ can be a nonnegative real number expressing the strength of the relation R between units X_i_ and X_j_.

One of the main procedural goals of blockmodeling is to identify, in a given network, clusters (classes) of units that share structural characteristics defined in terms of R. The units within a cluster have the same or similar connection patterns to other units. They form a clustering C={C_1_, C_2_,…, C_k_} which is a partition of the set

E: U_i_C_i_ = E and i ≠ j → C_i_ ∩ C_j_ = ∅. Each partition determines an equivalence relation (and vice versa). A clustering C partitions also the relation R into blocks R(C_i_,C_j_)=R ∩ C_i_ × C_j_. Each such block consists of units belonging to clusters C_i_ and C_j_ and all arcs leading from cluster C_i_ to cluster C_j_. In sum, a blockmodel consists of structures obtained by identifying all units from the same cluster of the clustering C [6,7].

Let’s introduce some details about the “direct” approach. The procedure is “to construct an appropriate criterion function directly and then use an optimization algorithm to obtain a clustering solution” [7], pg 461. In our case, the direct approach allows us to test our stylized vision on the intention of investment influenced by networks. The first step is to define a Criterion function P(C) that “has to be sensitive to the considered equivalence: P(C)=0⇔C . Following a give clustering C={C_1_, C_2_,…, C_k_}, let $\beta (C_{i},C_{j})=0$ denote the set of all ideal blocks corresponding to block R(C_i_,C_j_). In Pajek, this step consists in:

1. Determine the initial clustering C, while in the neighborhood of the current clustering C, there exists a clustering C’ that
a. $P(C^{\prime })<P(C)$. Do move to C’;
2. With practical consequence of this, move a unit X_k_ (a country, in our case) from cluster C_p_ to cluster C_q_, performing a transition process;
3. Performing a transposition interchanging countries from one cluster to another, always taking in account the criteria defined in the ideal block. [7] presents all the possibilities for building the “ideal block” based on the concept of equivalence.
4. After the results are: a) the new proposed block, with the relation between clusters and the countries inside each cluster; b) the accountability of mistakes, meaning links not preview in the ideal block, that remains after the optimization processes.

A comparison between two periods indicates changes in the organization of the network of flows of investment intentions. It is also possible to see a country’s changing role before and after a crisis. For instance, take the ideal block proposed to Pajek’ Block modeling/Operation section for manufacturing and for electricity sectors:

This ideal block in Table 1 above could be interpreted as follow:

**Table 1:** Ideal Matriz (image) proposed to the 1-Mode Blockemodeling.

	1	2	3	4
1	Regular	Regular	Null	Column Regular
2	Regular	Regular	Null	Column regular
3	Row Regular	Row Regular	Regular	Null
4	Null	Null	Null	Null

Source: the authors.

1. Four clusters are proposed: a center (core), an intermediate (sub-center), a satellite (to accommodate countries with a weird role), and the periphery.
2. The upper square in the table one looks to fit the relations between the center, intermediate (diagonal of the matrix), and the relations between center and intermediate and vice-versa.
3. Cluster 3 in the line points to the possibilities of the satellites investing in other blocks; satellites are not expected to receive investments from the center or intermediate, reflecting the null blocks in the columns.
4. Periphery do not invest in other countries but receive investments from center and intermediate countries;
5. After the clusters are identified using Louvain’s procedures, the relation of center, intermediate, and periphery receives better qualification.

## Analysis of the features of the networks

### Manufacturing: Structure and changes

The manufacturing network accounts for 124 countries. Taking the period before crisis as a reference in Table 2, the number of lines is 2084, the average degree is high, with 32 links per country, and the number of potential links is 12.8% of the total.

**Table 2:** Manufacturing networks characteristics before and after 2008 crisis.

Indicator class	Indicator	BEFORE	AFTER	variation %
**Exploratory**	**Number of Lines**	**2084**	**2373**	**+12.18**
**Cohesion**	**Density**	**0.13**	**0.15**	**+13.33**
**Cohesion**	**Average Degree**	**32**	**36**	**+11.11**
**Cohesion**	**Hierarchy**	**210**	**210**	**0.00**
**Cohesion (typical unexpected microstructure)**	300*;210*	**799;2248**	**1112;3510**	**Smooth relative reduction**
**Ranked possibilities**	120U*	**3510**	**3753**	**Smooth relative reduction**
**Ranked possibilities**	**Betweeness**	**0.09**	**0.11**	**+18.18**
**Center periphery**	**Centralization (in;out)**	**0.28;0.73**	**0.32;0.63**	**+12.5; - 15.87**
**Center periphery**	**Assortativity (in;out)**	**-0.11;-0.21**	**-0.10;-0.22**	**-10.00; + 4.55**

**Source: Author`s elaboration using FT data base and Pajek.**

The “Triad Census” reveals a particular and significative structure [16] with many triads. An expected triad of networks of this size (about 2000 lines) is a transitive circuit driving the intentions of investment in the manufacturing sector when country A invests in country B decide to invest in country C. Country C, by this side, invest in A. This is typical of “core” regions when assets are complementary between markets and firms. To a certain extent, each country has some specialization that creates opportunities in the other country. However, triad census has shown three unexpected microstructures in the network: a) 120U, which is typical of a ranked network, like those that represent genealogic trees; b) 300 and 210 show the presence of cliques, closed circuits that suggest the existence of intense flow of investments between countries in the manufacturing sector.

The in-betweenness centralization, 0.09, what is moderate, regarding the fact that is a direct network and there are 30 countries that only receive investment intentions - to reach those countries the path only goes through their donors. The center-periphery feature shows significant differences between the in and out closeness centrality index distribution. On one side, the in-closeness centrality indexes have a low coefficient of variation and a distribution that fits a normal distribution. The other out-closeness centrality index distribution is asymmetric to the left (median bigger than average), reflecting that even countries that receive investments, like China and Brazil, have many foreign investment projects. Table 2 shows that in general (all degrees), the closeness variation is high, 0.64, influenced by the out-closeness centrality. Finally, the indexes of assortativity confirms the asymmetry due to the hierarchy of the position of the countries in the map of countries who are investor and receivers, always recognizing the existence of almost 30 countries that do not provide any money to other countries. The out-in assortativity index is higher in absolute terms than in-out, meaning that countries that receive project intentions are similar that countries that send investments.

All of the date are coherent to a hierarchical structure and the possibility to apply a positional analysis (blockmodeling) to see the roles of countries in a world perspective.

The comparison with the pos-crisis is relevant to understand the resilience of the structure in manufacturing sector. Table 2 shows the slight increase of 12.2% in the numbers of lines after the crises, meaning the intention of increase projects of foreign investments; with the increase of cohesion, with a gain of 13.3% in the density index, profiting of the opportunities of connection. The average number of countries to whom each country is connected in average also grew.

The structure of the network remained the same after the crisis. The two indicators of the unexpected presence of triads (300 and 210) are still significant, in line with the expansion of the network, they are less expressive, showing a smooth relative reduction in their importance. The same happens with the index of ranking possibilities, 120U. After the crisis, there was a soft spread of the links. The increase in the betweenness confirms this, with a slight rise in reachable countries. The indexes of assortativity express the same phenomena in the two periods. A striking phenomenon is two complex networks in sixteen years, with a profound shock in 2008, which kept the same aggregate structure of FDI intentions.

The combination of positional analysis and cluster composition may better explain the changing environment and its effects on the network after 2008.

### Positional analysis: Before Crisis

The generalized blockmodeling approach allows blocks with distinct roles beyond a center-periphery approach [20]. The core comprises 12 countries, but only one was responsible for its existence: EUA, with projects towards 85% of the countries and receiving intentions only of 33% (confirming the differences between in and out assortativity indexes). China, India, and Emirates have formed triads with trilateral investment intentions. They were also net receivers. Spain was close to the EUA, receiving help from the integrated block and investing in some regions, mainly Latin America. The “core” has taken a priority look at integrated countries but was, in total, a net receiver, attracting funds from the integrated sector.

The integrated block was significant, comprising 59 countries. The net investors are the big European countries (Germany, The Netherlands, the UK, France), Japan, Korea, and Taiwan. The block contained the majority of microstructures described in the paper’s previous session (triads), resulting in the absorption of investment intentions inside it. Although there was a propensity to invest inside the block, their countries are providers to the core and periphery. The integrated block was the source of the structure’s resilience to the shocks.

Periphery accounted for 52 countries (42%), receiving less than 10% of the total intentions of investments and no investments abroad. It is worth highlighting the countries of Maghreb, Western Asia, and big countries of Africa, particularly Nigeria and some Central Asian countries. Regarding the sector’s importance in economic development, it is clear how difficult it is to change the productive structure of a relevant part of the world economy.

The positional analysis, via blockmodeling, as [31], shows the importance of an integrated block in the manufacturing sector, able to generate close circuits of investment (cliques) based on asset complementarity inside the productive chains, corporation affiliation, proximity, business tradition and positive effects of multilateral trade agreements.

### Positional analysis: After Crisis

The best blockmodeling fitting was basically the same as the one from the “before crisis” period. The structure is quite similar, with 12 countries in the core, 69 countries in the integrated block, and a reduction to 42 countries in the periphery, suggesting an inclusion process. The mentioned increase in the number of lines corresponds to more than US$ 300 billion in the volume of resources dedicated to foreign projects.

The “core” composition changed, and the block became a net investor with Germany’s participation. The Russian Federation received and invested in the USA. Other countries were net receivers linked to the USA and countries outside the block. After the crisis, the “core” better fitted the concept of a block that is intensively linked to other blocks, particularly the integrated block.

The integrated blocks became more significant and self-sufficient. However, despite including many countries from the periphery, the cluster is heterogeneous. It is worth pointing out the leading net investors: Japan, Korea, the UK, France, and Spain. China was relevant both as an investor and receiver. India was an emergent net receiver. The results confirm the emergence of Eastern Asia as a manufacturing pole.

The peripheral region reduced to 42 countries, receiving 8% of the total investment, without any relevance as investors. The manufacturing sector after the crisis has a selection of donors, the most relevant in the “core,” showing a redesign of the international composition of value chains. The integrated sector shows relative weakness in Europe and Latin America.

### Clusters

Fig 3 presents a graph of the foreign investment projects in manufacturing “before the crisis.” The Louvain algorithm without weights showed a low Q value, around 20%. Starting with the cluster that included EUA, the core’s Netherlands, France, Canada, and Spain were linked to Latin American countries, Mexico, and the periphery, like Ecuador, Honduras, Panamá, and El Salvador. Countries in the Middle East were included, but Saudi Arabia is present due to its strong link with the EUA. A vital cluster presented three Asian countries, India, Singapore, and Emirates, at the core, and the UK (leading donor) was in the integrated sector. They were linked to countries in Southern Asia, Maghreb, and Africa. The European cluster represented the idea of an “integrated block,” with Germany as the leading provider. It also includes some countries of the periphery in Europe, such as Bulgaria, Bosnia-Herzegovina, and Albania. This organization gives some clues to the factors that explain the expectations underlined in formulating investment projects: historical affinities, regional determination, asset complementarities, and the configuration of the value chains.

“After crisis” shows a similar number and composition of clusters. Following Fig 4, the most prominent cluster, led by Germany, has shown the most significant change regarding the previous period. Mexico, South Africa, and Canada are at the core, and many of the countries from the integrated sector, including some net investors like The Netherlands and Switzerland, are at the core. They are linked with the periphery of Latin and Central America, Mozambique, and Ghana. The cluster comprising the UK and France, plus some small European countries as providers, had an exact composition of a similar cluster before the crises: India, some Western Asian countries, and a large periphery composed of African and Southern Asian countries. Asian cluster is practically the same, except for the displacement of Korea as a provider, with the Russian Federation, Saudi Arabia, Iran, Malaysia, and Vietnam forming another cluster.

It is important to highlight that the value of the Q index is low, and the existence of the core means that some countries are net providers inside and outside its cluster. Fig 4 suggests a consolidation of regional clusters led by countries that kept their provider role, such as Japan, Germany, the United Kingdom, and Korea. The results also suggest two main subtle changes after the crisis: a) the spread of the network, bringing peripherical countries to the integrated sector, and, coherently, b) a regional organization with better integration between core and integrated sectors.

### Electric sector: Structure and changes

The Electricity sector accounts for 100 countries. According to Table 3, the number of lines before the crisis is 425, the average degree is low, 7.6, and the network is sparse. Few triads are concentrated in the 34 countries inside the strong component. The significative microstructures are a) 120U and 120D, typical of a ranked network. The first microstructure represents, for instance, two countries of the integrated block that send and receive the project’s intentions and both invest in a periphery country; b) 300, 120C, which show the presence of cliques, closed circuits that suggest the existence of the flow of investments between countries; c) 210 reveals the presence of asymmetry, the case when two countries reciprocate but a third one only send, not receiving from one of the remaining countries of the triad.

**Table 3:** Electricity networks characteristics before and after 2008 crisis.

Indicator class	Indicator	BEFORE	AFTER	variation %
**Exploratory**	**Number of Lines**	**425**	**692**	**+62.82**
**Cohesion**	**Density**	**0.04**	**0.06**	**+50.00**
**Cohesion**	**Average Degree**	**7.6**	**11.8**	**+55.26**
**Cohesion**	**Hierarchy**	**210**	**210**	**0.00**
**Cohesion (typical unexpected microstructure)**	300*;210*	**14;51**	**44;146**	**Cliques significative increase**
**Ranked possibilities**	120U*	**103**	**395**	**Cliques significative increase**
**Ranked possibilities**	**Betweeness**	**0.08**	**0.08**	**0.00**
**Center periphery**	**Centralization (in;out)**	**0.13;0.33**	**0.17; 0.41**	**+30.77; + 24.24**
**Center periphery**	**Assortativity (in;out)**	**-0.13; -0.11**	**-0.13; -0.12**	**0.00; + 9.09**

**Source: Author`s elaboration using FT data base and Pajek.**

The in-betweenness centralization, 0.08, is moderate because it is a direct network, and there are 66 countries out of the strong component. The path to those countries needs to go through their donors. Like the manufacturing sector, the Center-periphery feature causes significant differences between the in and out closeness centrality index distribution. Table 3 shows that in general (all degrees), the closeness variation is moderate, 0.45, reflecting the decentralized structure of the network (USA, for instance, is not a big player in this scene). The in-out and out-in assortativity indexes are similar, reflecting the high selectivity of the sector: investors and receivers are predominantly in the segment of strong components with heterogeneous in and out-degree values.

Table 3 shows an expressive increase in lines after the crisis, reflecting the diffusion of renewable energy sources and thermoelectric plants, which are modular projects. There is also an increase in cohesion, with more connections per country on average.

The structure of the network remained the same after the crisis. The two indicators of the unexpected presence of triads are still significant: a) 300, 120C, and 210 contribute to the increase of triads, representing cliques (circuits of investment), close circuits, and some transitivity; b) 120U and 120D contribute to the possibilities of definition of distinct roles of countries in the application of positional analysis.

Similar to what has occurred in the manufacturing sector, new countries are included in the strong components (de 34 to 27), representing a denser network structure with a broader reach. In the period after the crisis, the number of triads and close circuits of investment also increased.

### Positional analysis: Before Crisis

The model of generalized blockmodeling is almost the same as manufacture, aiming the comparability of the results: it looks for a definition of the four components: a) “core,” b) “integrated sector,” c)” satellite” and, d) “periphery”. However, unlike the manufacturing sector, the periphery has attracted investment from the core and integrated sectors.

The best-fit results were in 5 countries in the “core,” 17 in the “integrated sector,” 7 in the “satellite” (with no relevance), and 71 countries in the “periphery.” However, both core and integrated have invested in the periphery, performing a scheme similar to one presented in [20]: the core has sent almost half of its projects to the integrated system, and the latter, nearly 60% of their projects to periphery, resulting that these countries have received about 50% of all the electric intentions of investment during the period before the crisis.

Fig 5 shows that according to the results, the “core” Germany, Spain, China, the UK, and India are the first two net investors. The USA, Japan, France, Canada, and Italy were net investors driven their investments to the periphery. From Asia, Vietnam investment intentions in Laos and Cambodia. Malaysia, misplaced in the periphery by model fitting, has acted as an intermediary (broker) with foreign investments in Vietnam, Indonesia, Thailand, Laos, and Cambodia.

### Positional analysis: After Crisis

The network has become denser after the crisis, what is clear to see in Fig 6. The best-fit results have put 13 countries in the core, 13 in the integrated sector, 11 in the satellite (with no relevance), and 63 in the periphery. The average amount of money for projects in the period grew in all blocks after the crisis (36% to 66%). Despite the changes in composition, the main feature of the relationship between blocks remained: the periphery is a block that attracts investment projects, gathering 53% of the total amount of electricity projects.

The core is robust, with investment intentions for other blocks, particularly the periphery. China showed a stark change between the two periods, becoming a net investor when it directed its intentions to South Asia, particularly India and Pakistan. Japan, Germany, France, Korea, and the Russian Federation are net investors, some with direct links with the periphery (Russia to the Middle East) and others with investment intentions to developed countries of the integrated system, like the UK. The result, in line with the theory [20], is the change of the USA, receiving investment intentions from European countries and investing in the periphery.

The integrated sector shrinks a little, but the UK and India continue to be net receivers from the core that intend to invest in the periphery, Eastern Asia, and Southeast Asia countries. The agents of Spain, Italy, and Portugal have intended to invest in the integrated block and periphery. Malaysia moved to the integrated sector and invested in Eastern Asia (Indochina) countries. As in the period before the crisis, Vietnam, Pakistan, and Nigeria attracted investment intentions from the core and integrated sectors.

### Clusters

Fig 6 shows that, before clusters, the electric sector has had a clear regional component. Japan and Korea have pointed their investment intentions to Vietnam and other small countries of the Middle East and Southern Asia (India, placed in the core). At the core, Spain led investment intentions in Portugal (a net receiver at that period) and Latin American countries. France led a miscellaneous cluster that included Brazil, Poland, and Qatar in the periphery.

Germany, placed in the core, shares a cluster with many European countries that were predominantly investors with links with small European countries. Germany and Russia had an intense bilateral exchange of intentions of investment. However, Russia still fulfilled the role of provider of the periphery, linked to countries from Central Asia and the Middle East.

The USA is part of the integrated block and has a bilateral relationship with Spain at the core. The government has led a cluster with some periphery countries, like Pakistan and countries of the Middle East and Africa, suggesting a strategic component driving investment intentions. Canada had an intense relationship with Nigeria, which created a small cluster. Finally, China has led a cluster with Southeast Asia, including Taiwan, Hong Kong (providers), and some African countries. At that period, China was part of some cliques with an open side toward the periphery.

After the crisis (Fig 6) one cluster was strongly reinforced: the USA joined Spain, Italy, and Portugal in a web of bilateral relations (cliques) and put their lenses toward Latin American countries (Brazil included) and some small European countries. Japan cluster remained stable, with the one exception being the proximity of the Russian Federation to Asian countries.

The most crucial change is China’s attitude towards the electricity sector. In the second period, China became, in the view of the agents, a net investor, focusing on India, Pakistan, and Morocco in the periphery. Canada kept its relatively isolated position in a cluster with Nigeria. The Netherlands kept its position in the European cluster but intensified its links with Canada.

In sum, it is remarkable how the business intentions in the electricity sector intensified while maintaining practically the same positional and clustering situation, with exceptions related to significant changes in the role of EUA, China, and the Russian Federation.

## Conclusion

This paper presents an original approach, combining different clustering methods in one figure. One is the Louvain without weights (once everything is measured in terms of indegree) using the concept of modularity. The other is the positional analysis by blockmodeling. It could be considered a methodological innovation.

We clear answer our research questions in the “Analysis of the Features of the Networks” section. Among our substantial set of results, we also highlight: i) the 2008 crisis had a deep impact on the networks equilibrium, ii) it was substantially deeper in electricity than in manufacturing, which suggests that manufacturing web of investment projects was more resilient to exogenous shock, iii) global intentions of investment cohesion and core-periphery relations increased after crises in both sectors, iv) from the network indicators, there was more ties after crises, the network were smaller, the information reached easily, central countries importance in the expectation formation increased substantially in manufacturing but unchanged in electricity, the information to the central countries became shorter after crises, and the flow of expectation sent between dissimilar increases after crises.

Contextual factors and lagging effects of the great recession are among our next research questions.
